# Chemometrics of Wheat Composites with Hemp, Teff, and Chia Flour: Comparison of Rheological Features

**DOI:** 10.1155/2013/968020

**Published:** 2013-03-24

**Authors:** Marie Hrušková, Ivan Švec, Ivana Jurinová

**Affiliations:** Department of Carbohydrates and Cereals, Institute of Chemical Technology in Prague, Technická 5, 166 28 Prague 6, Czech Republic

## Abstract

The mixolab, a rheological device developed recently, combines approved farinograph and amylograph test procedures. Analysing wheat flour composites with hemp, teff, or chia in terms of all three mentioned rheological methods, correspondence of farinograph, and amylograph versus mixolab features was examined by principal component analysis. The first two principal components, PC1 and PC2, explained 75% of data scatter and allowed a satisfying confirmation of presumed relationships between farinograph or amylograph and mixolab parameters. Dough development time and stability were associated with gluten strength (C1 torque point) and also dough softening (mixing tolerance index) had a link to protein weakening (C1-C2 difference). In the second mentioned case, amylograph viscosity maximum and amylase activity (C3-C4) closeness was verified. Starch and starch gel properties during mixing (C3, C3-C2, and C4) affect dough viscosity (C1) and rheological behaviour (dough development time and stability). Another important finding is unequivocal distinguishing of the composite subsets (of hemp, teff, and chia ones) by the used rheological methods and statistical treatment of multivariable data.

## 1. Introduction

Addition of nontraditional components offers new possibility of wheat flour properties and could be performed by milling product from hemp, teff, and chia seeds. By the way, mentioned components also improve nutritional value of products owing to protein content and further positive ingredients for health.

*Hemp *(*Cannabis sativa*) is planted as two subspecies, namely, ssp. *culta* and ssp. *indica*. The latter is called hash hemp and belongs to forbidden raw material with respect to intoxicating substances production. Hemp flour composition depends on variety and planting locality and differs according to preparation and defatting. Protein, fat, and starch rates are known to be 30–33%, 7–13%, and approximately 40%, respectively. It contains a significant level of beta-carotene and vitamins B_1_ and E. Considering mineral component aspect, a benefit could be found in higher portion of iron and zinc [1]. Approximately two-thirds of hemp proteins is composed by edestin, belonging to low molecular weight globulins.

*Teff *(*Eragrostis tef*) is classified into cereal group of the Poaceae (Gramineae) family. As reported by Gomez and Gusta [2], main producer is Ethiopia with annual production of 1 million tons (20% of local cereals yield). Flat bread *injera* (*ingera*) dominates among other culinary treatments, and it is manufactured from thin fermented dough with a portion of wheat flour. Because of its tiny seeds, whole meal flour is characterised by high rate of coating layers and sprout, resulting into higher content of insoluble polysaccharides. Proteins have nongluten nature and owing to prevailing portion of prolamins belong to easily digestible ones. From a nutritional benefit viewpoint, high minerals content is cited (mainly iron, calcium, phosphorus, and copper) and B_1_ vitamin.

*Chia *(*Salvia hispanica* L.) is an annual herb of the Labiatae family, producing seeds which were one of the basic foods from the Aztec civilization. Now, the main producers belong to Argentine, Columbia, and Peru. The composition includes minerals, about 20% protein, 30–32% fat, 30–40% polysaccharides (insoluble fibre as the important portion), and 2-3% fructooligosaccharides [3]. The fatty acid of the oil was found to consist mostly of C16:0, C18:0, C18:2, and C18:3. Both forms (black and white) exhibit a high antioxidant activity due to the presence of phenolic compounds and tocopherols. Chia seeds have been approved by the EFSA and may be used as novel food ingredients in bakery products in the EU at maximum 5% [4].

A goal of the presented work was an investigation of farinograph and amylograph versus mixolab features comparability, performed by correlation and hierarchical cluster analyses. Torque points C1 and C2, describing mixing part of the Mixolab test, could have relationships to for example, farinograph dough development time or mixing tolerance index, while a difference C3-C4 should be correlated with amylograph viscosity maximum. Secondly, three tested wheat flour composite subsets (wheat flour with hemp, teff, or chia ones) should be distinguished by statistical means according the influences of alternative flour on rheological behaviour. For this purpose, principal component analysis was used, studying influences of alternative flour and addition level factors.

## 2. Materials and Methods

### 2.1. Materials

Wheat flour used as composites base was of commercial origin, obtained from industrial mill Delta Prague in 2010, and is characterised by low ash (0.59%) and quite high protein contents 12.97%. Hemp flour samples K1, K2, and K3 were produced also in the year 2010. The former two hemp flour samples were gained as conventional planting regime commodity (E. Citterbartová's company, Czech Republic). On the contrary, the sample K3 was prepared from hemp grain bred under bioregime conditions (Hanf & Natur, Germany).

Wheat flour replacement was selected on levels of 5%, 10%, 15%, and 20% identically for all three hemp materials.

Teff flour R1 and R2 samples were produced by Tobia Teff UK Ltd., and they could be identified according their white and brown botanical type. Wheat flour replacement ratios were 10, 20, and 30% for both teff types.

Chia flour Ch1 and Ch2 samples were prepared from conventionally and organically produced white and dark chia seeds, bought from Aida Organic and Country Life CZ companies, respectively. Chia seed samples originated in Mexico. With respect to EU legislation, substitution levels were chosen as 2.5% and 5.0% of wheat flour.

Mixed composites were signed by combination of alternative flour type and its addition level, for example, K2-15 means ratio of 85%/15% (w/w) of wheat and K2 hemp flour, respectively.

### 2.2. Methods

Using factor 5.7, protein content (PRO) was determined according to the Kjeldahl's method (ČSN ISO 1871). The farinograph and the amylograph test was performed following ČSN ISO 5530-1 and ICC 126/1, respectively. Observed features were dough development time and dough stability in min (DDE and DST, resp.) together with mixing tolerance index (MTI, farinograph unit); DDE and DST express “protein strength” at dough mixing and protein resistance against dough overmixing. From amylograph curve record, the amylograph viscosity maximum (AMY, amylograph unit) was considered; it has a relation to starch physical-chemical stage and it corresponds to amylases activity. On the mixolab, predefined protocol “Chopin+” was used, with the test settings corresponding to user's manual and published earlier [5]. The test settings combine kneading speed 80 min^−1^, kneader temperature 30°C, heating rate 2°C/min, and total analysis time 45 min. Course of wheat dough behaviour as well as the composite dough ones was described by five basic torque points (C1–C5, N·m) and four pair differences (C1-C2, C3-C2, C3-C4, and C5-C4, abbreviated as, e.g., C12).

For statistical treatment of measured data, the software Statistica 7.0 (Statsoft, USA) was used. Within the pairs hemp-teff, teff-chia, and hemp-chia composites, differences between recorded rheological feature means as well as their scatters similarity were tested by pair Student's *t*-test on *P* = 95%. By linear correlation analysis, correspondence between mixolab versus farinograph and amylograph data was explored (*P* = 95%, 99%, and 99.9%). For variables closeness examination, both principal component analysis (PCA) and hierarchical clustering were employed. Histogram, the second method output, was built in Euclidean space according to the farthest neighbourhood clustering algorithm.

## 3. Results and Discussion

### 3.1. Properties of Tested Flour Composites

#### 3.1.1. Protein Content

Composite K1 and K2 subsets are characterised by PRO slightly higher than common level of the Czech wheat, that is, from 13.11 to 14.82% for samples with 5% and 20% of hemp flour. Blends containing hemp K3 type were somewhat richer in the component; determined limits were 13.46% and 15.94% for adequate blends. Pure teff flours R1 and R2 had the lowest PRO between tested nontraditional materials; with respect to this, their addition did not meant a nutritional benefit—in the former case, PRO oscillated around 12.57% and around 13.06% in the latter. According to smallest amount added (2.5% and 5.0%), wheat flour enhanced by chia ones demonstrated very slight increase of PRO from 10.97% to 11.21%, respectively, independently to Ch1 or Ch2 type.

#### 3.1.2. Rheological Characteristics

Farinograph data in Table 1 show a diverse influence of tested alternative flour types on wheat composite baking quality. Shorter the DDE and the DST times were observed only in cases of K1 and teff supplements, independently to their white/brown type. Such baking quality change meant also an increase in the MTI values, which were verifiably the highest just for R1 and R2 samples. Furthermore, chia mixtures could be clearly distinguished from the hemp ones in the DST (approximately twofold within the chia subsets).

During the amylograph test, viscosity of flour suspensions containing both tested chia types was recorded on approximately half level than other composites. Comparing hemp and teff subsets, suspensions viscosity maximum increased in the former and reversely diminished in the latter case as addition level of alternative flour increased (Table 1).

#### 3.1.3. Mixolab Profile

Interpretation of Mixolab curves determined in “Chopin+” measurement mode was published previously [5–7]. The first two mixolab curve parameters, C1 and C2, are related to protein baking strength. According to C1 and C2 torque points, analysed composite subsets could be partially and completely distinguished (variability a-b and a- c in Tables 1 and 2, resp.). During chia-dough mixing, the highest resistance and the lowest damage of protein structures was provably revealed correspondingly to their DST values. On the other hand, gluten-like proteins in both teff flour samples were the weakest (Table 2).

Indirect examination of anylases activity is represented by further three torque parameters, C3 as well as C4 during a heating and C5 during a cooling phase of the mixolab test. ANOVA results in Table 2 summarise broader extent of alternative flours influence—the strongest impact was significantly determined for both chia Ch1 and Ch2 samples.

### 3.2. Statistical Analysis

#### 3.2.1. Correlation Analysis

With respect to the paper aim, Pearson correlation matrix (as an exploration method) was calculated firstly. Considering three probability levels (of 95, 99, and 99.9%), the farinograph test is satisfyingly represented by the DST and the MTI features (Table 3). No significant relationships between the DDE and the Mixolab features could be explained by reversal hemp and teff versus chia effect on this dough property. The same reason (due to inconsistency within, e.g., hemp subset) leads to only one significant relationship of the AMY and the C1 (*r* = −0.46, *P* = 95%).

According to international standards, amylases activity could be described by the reference amylograph test, so by the screening method “Falling Number”. Codină et al. [7] mentioned tight association of the Falling Number with starch gelatinization C3, amylolytic activity C4, and starch retrogradation C5 (*r* = 0.877, 0.794 and 0.907) and their differences (C32 and C54, *r* = 0.878 and 0.953, resp.) within the set of 60 Romanian bread flour samples.

#### 3.2.2. Principal Component Analysis (PCA)

 As known, data for the PCA must be intercorrelated and observed subsets should be characterised by a similar data variance. Links among all 13 determined variables were presented above, and subset scatter comparability was tested by the pair Student's *t*-test. Measured feature averages of hemp-teff, hemp-chia, and teff-chia were not different in 9 cases, and dissimilar subset scatters were found in 11 cases of 39 in total (data not shown). Summarised, the PCA method could explain variability within the set correctly.

**Figure 1:**
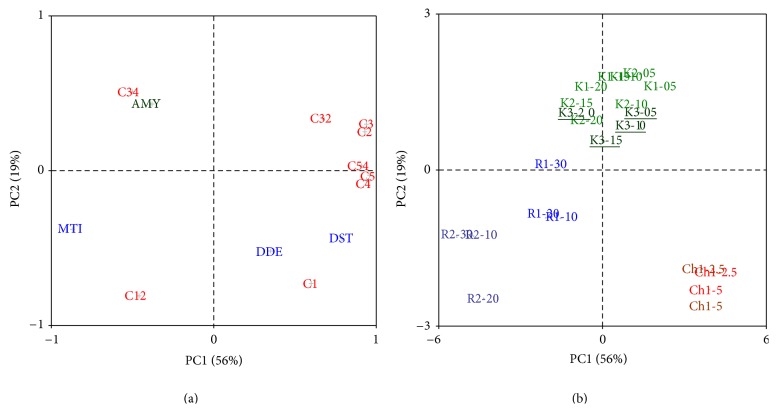
Comparison of rheological behaviour of wheat-hemp (K1, K2, K3), wheat-teff (R1, R2), and wheat-chia (Ch1, Ch2) composite flour by PCA. (a) variables loading plot—farinograph data: DDT: dough development time, DST: dough stability, MTI: mixing tolerance index; amylograph data: AMY: viscosity maximum; Mixolab torque data: C1–C5, and their pair differences C12, C32, C34, and C54. (b) Samples score plot.

Data variance was explained from 89% by the first three principal components (PCs), from 56%, 19%, and 14% by PC1, PC2, and PC3, respectively (Table 4). The mixolab and farinograph mixing parameters were associated together—along the PC1 axis, groups of DDE-DST-C1 as well as MTI-C12 represent the initial kneading phases of and dough consistence diminishing during the tests (Figure 1(a)). Connected also to the PC1, grouping of AMY-C34 and their link to torque points C4-C5-C54 reflects amylases activity and consequences of their action altogether. The third features combination is remarkable for DDE-DST and also five Cs as well as C32 and C54—enzymatic starch degradation results into dough resistance weakening. The findings correspond to conclusions mentioned in [7]—C1 had a link to dough stability during mixolab test in “Simulator” regime. Authors furthermore documented on the PC1 × PC2 plot, and C3, C4, C5 together with C32 and C54 were associated with the Falling Number testing.

Studying effect of composites chemical constitution, also all three tested subsets were successfully distinguished by combination of three employed rheological tests and the multivariable explorative statistics. A dominant influence of tested alternative crop is unequivocal in the PC1 × PC2 plot, over the crops conventional/organic origin, white/brown type or added amount factors (Figure 1(b)). As discussed previously, for example, hemp and chia composites differed in PRO and DST or estimated amylolytic activity (AMY).

#### 3.2.3. Hierarchical Clustering

**Figure 2:**
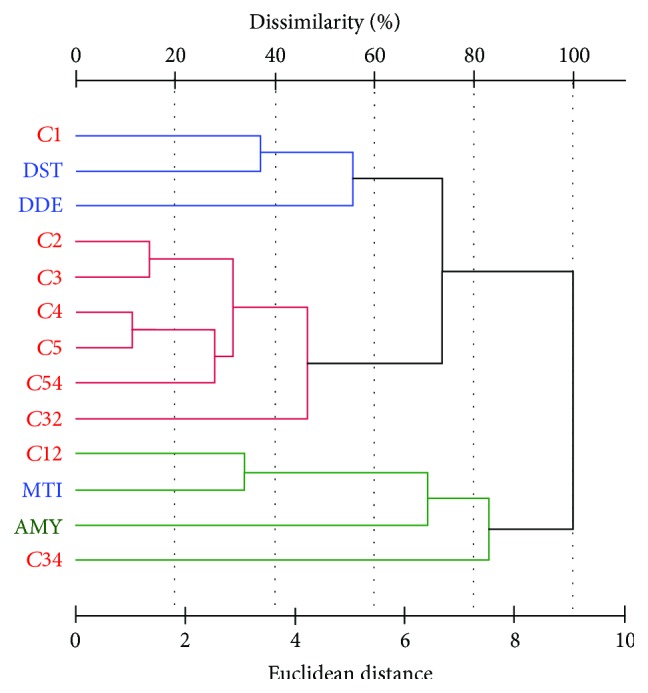
Cluster analysis between farinograph, amylograph, and mixolab parameters. Farinograph data: DDT: dough development time, DST: dough stability, MTI: mixing tolerance index; amylograph data: AMY: viscosity maximum; Mixolab torque data: C1–C5, and their pair differences C12, C32, C34, and C54.

Possible substitutions within the rheological feature groups were further tested by cluster analysis. Based on data pretreated by standardisation (due to diverse scales), a histogram was built in the Euclidean space using the farthest neighbourhood algorithm. Within Figure 2, three subunits could be identified in accordance with the PCA method results (e.g., DDE-DST-C1 in blue). For primary pair clusters (C1-DST, C12-MTI, C2-C3, and C4-C5), the features statistical similarity was determined at least 60%. To summarise, a distance of 9.07 units (100% dissimilarity) was evaluated between the MTI and the C2 rheological features.

## 4. Conclusions

Presented study experimental design included comparison of three rheological methods result, assessed for wheat flour blends with hemp, teff, and chia, and the PCA method used for features closeness determination as well as discrimination of the three tested composite subsets. Farinograph, amylograph, and mixolab data have shown basic differences in composite dough rheological behaviour, that is, in a bakery potential of these mixtures.

Correlation analysis partially signified some links between parameters of these three tests, and principal component analysis confirmed presumed relationships in agreement with conclusion published by other authors. Within 75% of explained variability (PC1 × PC2 plane) and considered farinograph characteristics, tight associations were revealed out between dough development time or stability and C1, and between mixing tolerance index and the difference C1-C2. Amylograph viscosity maximum was connected with C3, C4 and C5 torque points (and also with C3-C2, C3-C4, and C5-C4 differences), which correspond to amylases activity and starch gel properties during heating and cooling phase of the mixolab test. Levels of those torque points (C3, C4, and C5) corresponded to dough consistency changes (i.e., resistance against mixing), so relationships to farinograph dough development time and stability.

All these findings were confirmed by hierarchical clustering in Euclidean space, because mentioned features groups were found in built histogram.

## Figures and Tables

**Table 1:** Farinograph and amylograph test results of wheat flour composites.

Sample	Farinograph						Amylograph	
	DDE (min)		DST (min)		MTI (FU)		AMY (AU)	
(A) Effect of 5 and 20% of hemp								

K1-05	6.00	a	8.50	a	30	a	490	a
K1-20	5.50		11.50		40		365	
K2-05	3.00		10.00		30		500	
K2-20	8.00		11.00		45		380	
K3-05	3.50		8.00		40		565	
K3-20	6.00		7.00		50		680	

(B) Effect of 10 and 30% of teff								

R1-10	9.50	a	11.00	a	60	b	400	
R1-30	5.00		6.50		120		620	
R2-10	7.50		9.25		140		350	
R2-30	1.75		3.00		140		510	a

(C) Effect of 2.5 and 5.0% of chia								

Ch1-2.5	7.50	a	19.00	b	20	a	260	
Ch1-5.0	9.00		19.00		20		260	
Ch2-2.5	8.0		20.00		40		310	
Ch2-5.0	11.0		20.00		40		330	b

DDE—dough development, DST—dough stability, MTI—mixing tolerance index; FU—farinograph unit.

AMY—amylograph viscosity maximum; AU—amylograph unit.

a-b: column means related to alternative crop signed by same letter are not significantly different (*P* > 0.05).

**Table 2:** Mixolab test results of wheat flour composites.

Sample	Mixolab									
	C1 (N·m)		C2 (N·m)		C3 (N·m)		C4 (N·m)		C5 (N·m)	
(A) Effect of 5 and 20% of hemp										

K1-05	0.98	a	0.50	b	2.01	b	1.59	b	2.42	b
K1-20	0.95		0.49		1.91		1.25		1.86	
K2-05	1.02		0.51		2.03		1.56		2.17	
K2-20	0.94		0.46		1.90		1.32		1.84	
K3-05	1.06		0.48		1.99		1.60		2.34	
K3-20	0.94		0.41		1.80		1.41		2.08	

(B) Effect of 10 and 30% of teff										

R1-10	1.02	a	0.37	a	1.79	a	1.26	a	1.71	a
R1-30	0.99		0.26		1.79		1.32		1.88	
R2-10	0.93		0.20		1.58		0.94		1.44	
R2-30	1.04		0.21		1.57		1.12		1.47	

(C) Effect of 2.5 and 5.0% of chia										

Ch1-2.5	1.21	b	0.58	c	2.05	b	1.75	c	2.55	c
Ch1-5.0	1.19		0.53		2.00		1.69		2.57	
Ch2-2.5	1.16		0.54		2.01		1.72		2.53	
Ch2-5.0	1.22		0.56		2.03		1.68		2.52	

C1–C5: Mixolab torque data.

a–c: column means related to alternative crop signed by same letter are not significantly different (*P* > 0.05).

**Table 3:** Correlation analysis between mixolab, farinograph and amylograph test parameters.

Parameter	C1	C2	C3	C4	C5	C12	C32	C34	C54
DDE	ns	ns	ns	ns	ns	ns	ns	ns	ns
DST	0,73∗∗∗	0,68∗∗∗	0,63∗∗	0,63∗∗	0,67∗∗∗	ns	ns	ns	0,65∗∗
MTI	ns	−0,96∗∗∗	−0,93∗∗∗	−0,76∗∗∗	−0,79∗∗∗	0,77∗∗∗	−0,57∗∗	ns	−0,76∗∗∗
AMY	−0,46∗	ns	ns	ns	ns	ns	ns	ns	ns

C1–C5, and their pair differences C1-C2 (C12), C3-C2 (C32), C3-C4 (C34), C5-C4 (C54)—mixolab torque data.

DDE—dough development, DST—dough stability, MTI—mixing tolarance index.

AMY—amylograph viscosity maximum.

^∗,  ∗∗,  ∗∗∗^relationships significant at P = 95%, 99% and 99.9%, respectively; ns—non-significant.

**Table 4:** Proportion (%) of explained variation by first three principal components (PC's).

Parameter	PC1	PC2	PC3
C1	36∗	53∗∗	3
C2	87∗∗	6	3
C3	88∗∗	9	0
C4	88∗∗	0	11
C5	92∗∗	0	6
C12	21∗	65∗∗	10
C32	45∗∗	11	6
C34	25∗	26∗	43∗∗
C54	83∗∗	0	1

DDE	12	27∗	29∗
DST	62∗∗	19∗	15∗
MTI	76∗∗	14	2
AMY	14	19∗	53∗∗

Total	56	19	14

C1—C5, and their pair differences—mixolab torque data.

DDE—dough development, DST—dough stability, MTI—mixing tolerance index, AMY—amylograph viscosity maximum.

^∗,  ∗∗^singnificant pair correlationship between quality parameter and proper principal component.
